# High‐Entropy Metal–Organic Frameworks and Their Derivatives: Advances in Design, Synthesis, and Applications for Catalysis and Energy Storage

**DOI:** 10.1002/advs.202411175

**Published:** 2024-12-12

**Authors:** Jiangyuan Xing, Yan Liu, George Mathew, Qiu He, Jasmin Aghassi‐Hagmann, Simon Schweidler, Ben Breitung

**Affiliations:** ^1^ Institute of Nanotechnology Karlsruhe Institute of Technology (KIT) Kaiserstraße 12 76133 Karlsruhe Germany

**Keywords:** catalysis, energy conversion, energy storage, high‐entropy, metal–organic frameworks

## Abstract

As a nascent class of high‐entropy materials (HEMs), high‐entropy metal–organic frameworks (HE‐MOFs) have garnered significant attention in the fields of catalysis and renewable energy technology owing to their intriguing features, including abundant active sites, stable framework structure, and adjustable chemical properties. This review offers a comprehensive summary of the latest developments in HE‐MOFs, focusing on functional design, synthesis strategies, and practical applications. This work begins by presenting the design principles for the synthesis strategies of HE‐MOFs, along with a detailed description of commonly employed methods based on existing reports. Subsequently, an elaborate discussion of recent advancements achieved by HE‐MOFs in diverse catalytic systems and energy storage technologies is provided. Benefiting from the application of the high‐entropy strategy, HE‐MOFs, and their derivatives demonstrate exceptional catalytic activity and impressive electrochemical energy storage performance. Finally, this review identifies the prevailing challenges in current HE‐MOFs research and proposes corresponding solutions to provide valuable guidance for the future design of advanced HE‐MOFs with desired properties.

## Graphical abstract

High‐entropy metal‐organic frameworks (HE‐MOFs), as an emerging member of a high‐entropy family, have attracted considerable research interest as promising multifunctional materials. This review comprehensively summarizes the latest advancements in functional design, synthesis strategies, and versatile applications of HE‐MOFs. Additionally, an outlook on the prevailing challenges in HE‐MOFs research and corresponding solutions is provided to guide the rational design of advanced HE‐MOFs.

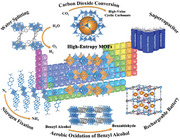

## 1 Introduction

Over the past few decades, global demand for energy and chemicals has experienced exponential growth due to the rapid development of modern society and the increasing population.^[^ ^1^, ^2^, ^3^ ^]^ Currently, the supply of energy and chemical raw materials is predominantly reliant on coal, crude oil, and natural gas.^[^ ^4^, ^5^ ^]^ However, the excessive consumption of these traditional fossil fuels not only leads to resource depletion but also emits greenhouse gases upon combustion, resulting in severe environmental challenges such as climate change and global warming.^[^ ^6^, ^7^, ^8^ ^]^ To address these challenges, it is imperative to explore sustainable and eco‐friendly alternatives. One crucial aspect involves the development of advanced energy storage technologies, such as rechargeable batteries and supercapacitors, to facilitate the seamless integration of intermittent renewable energy sources like solar and wind energy. Additionally, it is essential to design efficient catalysts to enable the high‐value conversion of naturally abundant and renewable resources, including biomass energy, water, carbon dioxide, and nitrogen, into high‐energy fuels and functional chemicals.

The successful implementation of the aforementioned applications heavily relies on the design of functional materials. Metal–organic frameworks (MOFs), constructed by the coordination of metal ions or clusters with organic ligands,^[^ ^9^, ^10^, ^11^ ^]^ have received significant research interests as promising multifunctional materials. With their inherent merits of large specific surface area, high porosity, and tunable chemical composition at the atomic level,^[^ ^12^, ^13^, ^14^, ^15^, ^16^, ^17^ ^]^ MOFs have been intensively studied in the fields of gas storage,^[^ ^18^, ^19^, ^20^, ^21^, ^22^ ^]^ electrochemical energy storage,^[^ ^23^, ^24^, ^25^, ^26^, ^27^, ^28^, ^29^, ^30^, ^31^ ^]^ and catalysis.^[^ ^32^, ^33^, ^34^, ^35^, ^36^ ^]^ However, despite demonstrating versatile applications, there remains substantial potential for enhancing both structural and chemical properties of MOFs to promote their performance in electrochemical energy storage and catalysis.^[^ ^37^, ^38^, ^39^, ^40^, ^41^ ^]^ Fortunately, the extension of the high‐entropy strategy from inorganic materials to organic‐inorganic materials has provided a new direction for improving their physicochemical properties.

**Figure 1:**
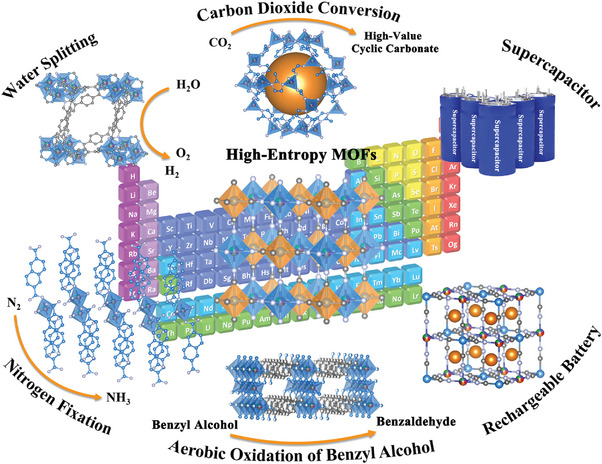
Schematic illustration of HE‐MOFs for catalysis and energy storage applications.

The concept of high‐entropy originated from two independent reports on multi‐element alloys by Yeh et al.^[^ ^42^ ^]^ and Cantor et al.^[^ ^43^ ^]^ in 2004. Since then, the high‐entropy strategy has progressively extended to a broader range of material systems, leading to the successful synthesis of various high‐entropy materials (HEMs), including high‐entropy alloys (HEAs),^[^ ^44^, ^45^, ^46^, ^47^, ^48^ ^]^ high‐entropy ceramics (HECs),^[^ ^49^, ^50^, ^51^, ^52^, ^53^ ^]^ high‐entropy sulfides (HESs),^[^ ^54^, ^55^, ^56^, ^57^ ^]^ high‐entropy phosphides (HEPs),^[^ ^58^, ^59^, ^60^ ^]^ HE‐MOFs,^[^ ^61^, ^62^, ^63^, ^64^ ^]^ and others. As a new member of HEMs family, HE‐MOFs and their derivatives have achieved rapid developments in various research areas due to their controllable chemical functionalities, abundant active metal sites, stable porous structure, and enhanced conductivity (**Figure** **1**). In the field of catalysis, the uniform distribution of multiple elements in HE‐MOFs can create numerous active sites, while their high porosity facilitates contact between catalysts with intermediates, thereby improving the efficiency of adsorption/desorption processes.^[^ ^65^, ^66^, ^67^ ^]^ When used as electrodes for rechargeable batteries, their stable framework structures can provide wide channels for the insertion and extraction of large‐size metal ions like Na^+^, K^+^, and Zn^2+^ and enhance cycling stability. Additionally, the cocktail effect of multiple cations integrated into HE‐MOFs can greatly improve their ion storage capacity.^[^ ^68^, ^69^, ^70^ ^]^

However, up to now, there has been a lack of a comprehensive review summarizing the recent progress of emerging HE‐MOFs. Consequently, the main objective of this article is to provide a detailed overview of the latest advancements in synthesis strategies, chemical properties, and versatile applications of HE‐MOFs. Furthermore, we intend to emphasize their remarkable performance in the fields of catalysis and electrochemical energy storage. In addition, the challenges and perspectives for the future developments of HE‐MOFs are also discussed, providing novel guidance for designing advanced HE‐MOFs.

## 2 Definition and Properties of HE‐MOFs

HEAs are the earliest reported HEMs, which refer to alloys containing five or more elements at molar ratios ranging from 5%–35%.^[^ ^42^ ^]^ Subsequently, with the rapid development of the high‐entropy concept, HEMs have been more quantitatively defined as materials with configurational entropy (∆*S*_conf_) > 1.5 R, where *R* represents the ideal gas constant. Accordingly, MOFs, with a single disordered sublattice, can be classified as high‐entropy MOFs, medium‐entropy MOFs (ME‐MOFs), and low‐entropy MOFs (LE‐MOFs) when their ∆*S*_conf_ ≥ 1.5 *R*, 1.0 *R* ≤ ∆*S*_conf_ < 1.5 *R*, and ∆*S*_conf_ < 1.0 *R*, respectively.^[^ ^71^ ^]^ Generally, for a disordered n‐components MOF, its ideal configurational entropy can be calculated using the following Equation 1:

(1) $$\Delta S_{\mathrm{conf}}=-R\sum_{i=1}^{n}x_{i}\ln x_{i}$$

where *n* is the number of elements, and x_i_ indicates the mol fraction of the i^th^ component.

**Figure 2:**
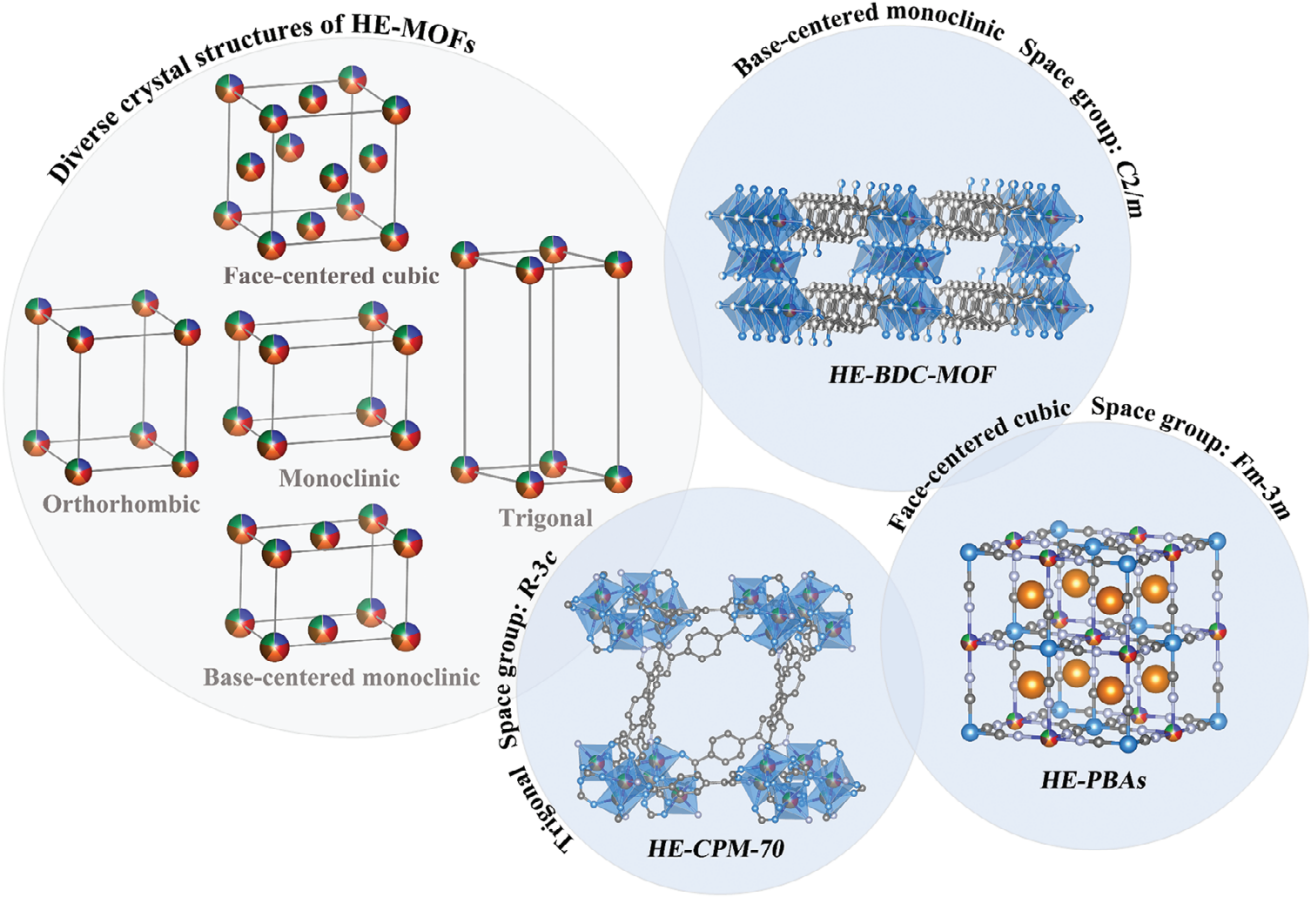
Diverse lattice structure models (left), and some corresponding crystal structures (right) of HE‐MOFs.

Until now, various HE‐MOFs with distinct crystal structures, including face‐centered cubic, monoclinic, base‐centered monoclinic, orthorhombic, and trigonal, have been successfully synthesized (**Figure** **2**).^[^ ^27^, ^61^, ^62^, ^72^, ^73^ ^]^ Compared to conventional MOFs, HE‐MOFs possess three unique effects, including lattice distortion,^[^ ^61^, ^74^ ^]^ sluggish diffusion,^[^ ^75^ ^]^ and cocktail effect,^[^ ^69^, ^76^ ^]^ collectively contributing to the attractive properties of HE‐MOFs. Specifically, lattice distortion results from stochastic occupation of the lattices by cations with different ionic radius and electronegativity, impacting the chemical, physical, and mechanical properties of HE‐MOFs. In the field of electrocatalysis, for example, the slight lattice distortion in certain HE‐MOFs plays a crucial role in changing the coordination environment around metal species, which affects the binding energy between active sites and intermediates, ultimately enhancing the catalytic activity.^[^ ^72^ ^]^ The presence of sluggish diffusion leads to the formation of HE‐MOFs with amorphous or nanocrystal phases, which enhances the electrocatalytic activity for water splitting due to the existence of abundant defects.^[^ ^75^ ^]^ Interestingly, in contrast to the slow diffusion kinetics observed in HEAs due to substantial lattice potential energy fluctuations caused by lattice distortion, most findings suggest that the diffusion of cations within HE‐MOFs is comparable to or even faster than in non‐HE‐MOFs. This is evidenced by the superior rate capability and higher cation diffusion coefficient observed in HE‐MOFs electrodes. Finally, the cocktail effect is used to describe the attractive but unpredictable properties of HE‐MOFs. Generally, the distinctive properties of HE‐MOFs do not arise from a simple summation of the properties by their constituent components, but rather from the cooperative interactions between the different metal ions with versatile chemical features. For instance, when utilized as cathodes in rechargeable batteries, the cocktail effect of HE‐MOFs is conducive to enhancing their electrochemical performance.^[^ ^50^, ^77^ ^]^

## 3 Synthesis Strategies of HE‐MOFs

According to the Gibbs free energy formula (Equation 2), there is a competitive relationship between ΔH_mix_ (the enthalpy of mixing) and ΔS_mix_ (the entropy of mixing). A system will achieve thermodynamic equilibrium when its Gibbs free energy is minimized at a specific pressure and temperature. Generally, a higher number of elements can increase the ΔS_mix_, which potentially reduces the Gibbs free energy. However, the absolute value of ΔH_mix_ is typically much larger than that of ΔS_mix_, making ΔH_mix_ often act as the decisive factor. As a result, entropy stabilization, where TΔS_mix_ overcompensates for ΔH_mix_, occurs only under high‐temperature conditions. This thermodynamic condition can promote the formation of a single‐phase solid solution. Therefore, theoretically, high‐entropy lattices can be achieved by heating reactants to high temperatures.

(2) $$\Delta G_{\mathrm{mix}}=\Delta H_{\mathrm{mix}}-T\Delta S_{\mathrm{mix}}$$

However, over the years, research on HEMs has revealed that achieving a truly disordered mixing of different elements through traditional solid‐state reaction routes is challenging. This difficulty arises from the presence of numerous local energy minima during the reconstruction of high‐entropy lattices, which correspond to thermodynamically metastable states but are mistakenly regarded as high‐entropy states due to kinetic constraints.^[^ ^78^ ^]^ Moreover, high synthesis temperatures in solid‐state approaches further limit a broader utilization of high‐entropy organic‐inorganic hybrid compounds. Consequently, HE‐MOFs are commonly synthesized via a bottom‐up strategy under ambient or low temperatures. In contrast to solid‐state synthesis of HEMs like high‐entropy oxides (HEOs), this bottom‐up strategy enables uniform mixing of various elements in precursor solutions and eliminates the requirement for high synthetic temperatures to release different metal ions from their original lattices. Furthermore, incorporating organic linkers into the HE‐MOFs lattices increases the distance between different metal species compared to HEOs, and modifies the electronic environment of individual metals, such as spin states. These differences reduce the degree of lattice distortion and weaken the impact of Δ*H*_mix_. Therefore, these underlying mechanisms allow for the atomically precise construction of high‐entropy frameworks at low temperatures, achieving a balance between thermodynamic preference and kinetic feasibility. Up to now, various chemical techniques, including solvothermal/hydrothermal methods, co‐precipitation, wet chemistry‐assisted mechanochemistry, and electrodeposition approach have been reported for synthesizing HE‐MOFs with homogeneous elemental distribution.^[^ ^27^, ^61^, ^66^, ^79^, ^80^ ^]^

### 3.1 Solvent‐Based Metal Incorporation

**Figure 3:**
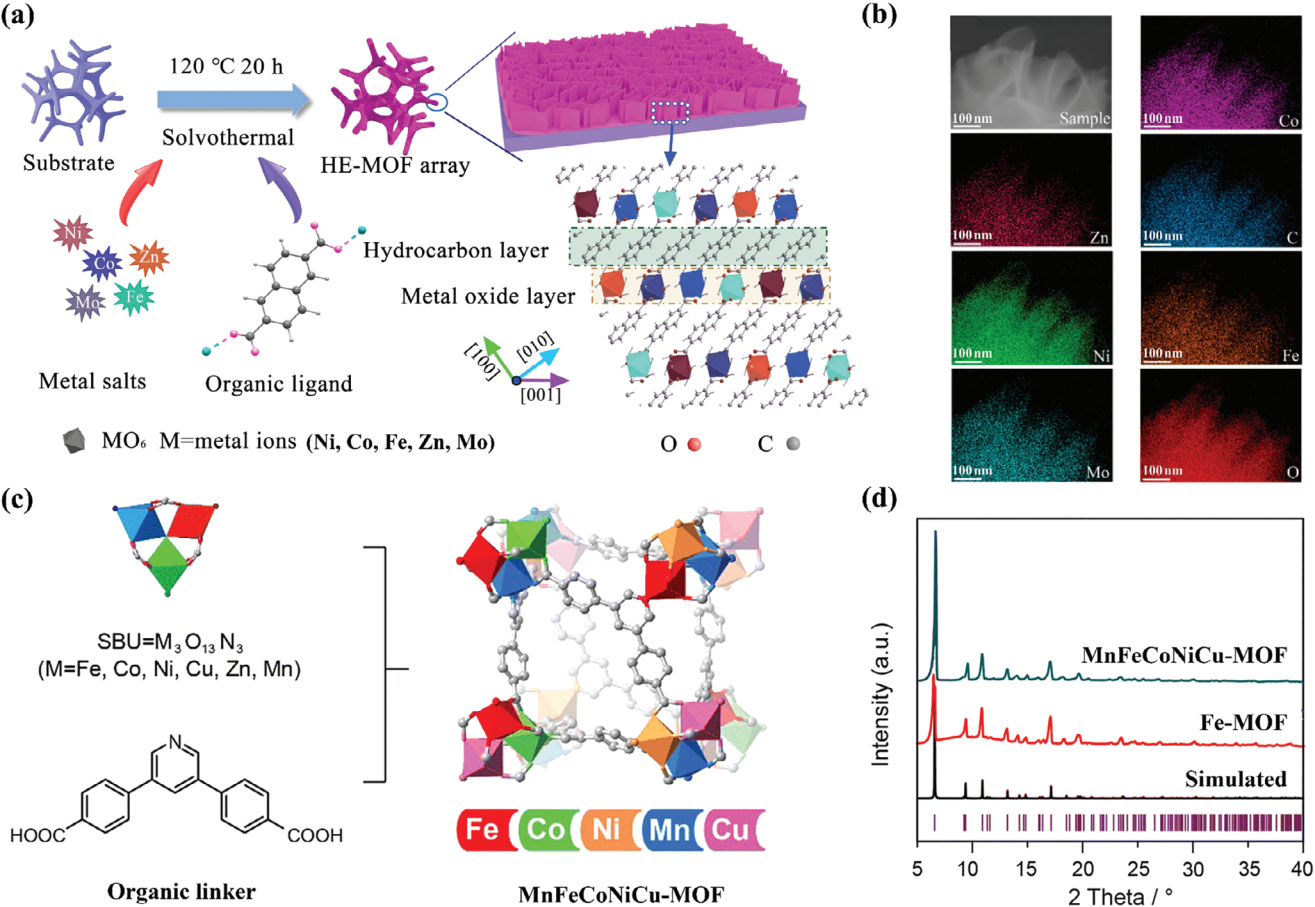
a) Schematic illustration of the synthetic process and b) EDS maps of HE‐MOF array. Reproduced with permission.^[^ ^81^ ^]^ Copyright 2022, American Chemical Society. c) Schematic illustration of the construction of MnFeCoNiCu‐MOF using SBUs and organic linker. d) The XRD patterns of MnFeCoNiCu‐MOF and Fe‐MOF. Reproduced with permission.^[^ ^73^ ^]^ Copyright 2022, Royal Society of Chemistry.

As commonly employed approaches to obtain HE‐MOFs, solvothermal and hydrothermal methods typically involve heating a well‐mixed solution of multiple metal salts and organic ligands under low‐temperature and high‐pressure conditions, which ensures sufficient energy input while avoiding the destruction of organic‐inorganic hybrid structure caused by high temperature (>400 °C). As shown in **Figure** **3**a, utilizing the solvothermal approach, Xu et al. fabricated HE‐MOF by introducing five metal elements with similar ionic radii to coordinate with the organic ligands 2,6‐naphthalenedicarboxylate tetrahydrate.^[^ ^81^ ^]^ The obtained HE‐MOF displayed a vertically aligned array structure, which effectively suppresses nanoparticle restacking and promotes the formation of hierarchical porosity, facilitating the transfer of reactants and gas bubbles. Additionally, the energy‐dispersive X‐ray spectroscopy (EDS) maps of HE‐MOF (Figure 3b) demonstrated Ni, Co, Fe, Zn, and Mo are uniformly dispersed on the nanosheets with near‐equimolar ratios. Using similar solvothermal methods, also other high‐entropy MOFs can be synthesized. Yan et al. adjusted such a method to synthesize a series of multivariate MOFs. Specifically, as presented in Figure 3c, various metal ions were initially combined with trifluoroacetate to form trinuclear metal oxide clusters, acting as secondary building units (SBUs) to enhance the compatibility of various transition metals within the framework structures.^[^ ^73^ ^]^ These SBUs were then assembled with organic linker 4,4′‐(pyridine‐3,5‐diyl)dibenzoic acid to generate MnFeCoNiCu‐MOF. The X‐ray diffraction (XRD) pattern of MnFeCoNiCu‐MOF revealed a single‐phase trigonal structure, which matches well with the simulated one (Figure 3d). Moreover, the EDS results confirmed the homogeneous distribution of different elements within this material. As a special subclass of solvothermal methods, hydrothermal approaches could also be applied for HE‐MOF synthesis. Zhou et al. reported the preparation of Ni foam‐supported CoNiFeMuCu‐based HE‐MOF via the hydrothermal method.^[^ ^66^ ^]^ They dissolved a series of metal salts and 2,5‐dihydroxyterephthalic acid in a solvent mixture of *N,N*‐dimethylformamide, ethanal, and water. Then, the HE‐MOF with uniform elemental dispersion was obtained by heating this solution at 120 °C for 24 h.

### 3.2 Co‐Precipitation

**Figure 4:**
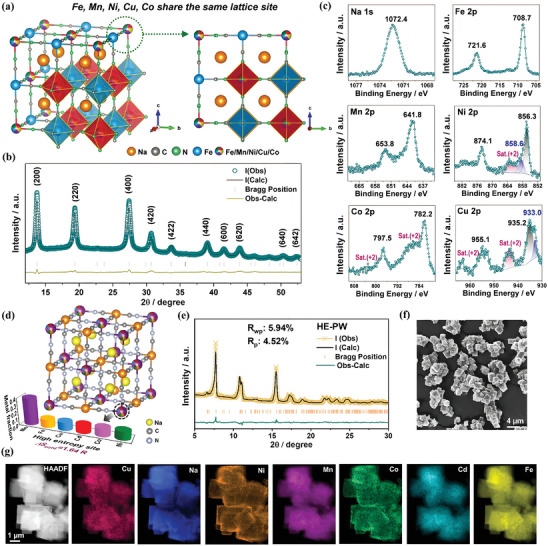
a) Schematic illustration of the crystal structure, b) XRD pattern and corresponding Rietveld refinement profile, and c) high‐resolution XPS spectra of Na 1s, Fe 2p, Mn 2p, Ni 2p, Co 2p, and Cu 2p of HE‐PBA. Reproduced with permission.^[^ ^69^ ^]^ Copyright 2021, Wiley‐VCH. d) Schematic illustration of the crystal structure, e) powder XRD pattern and corresponding Rietveld refinement profile, f) SEM image, and g) EDS maps of HE‐PW. Reproduced with permission.^[^ ^68^ ^]^ Copyright 2023, Wiley‐VCH.

Another interesting synthetic approach toward HE‐MOFs is based on co‐precipitation methods. Compared to the previously discussed solvothermal and hydrothermal methods, this approach offers an advantage by eliminating the need for external energy input or heating, allowing for the preparation of nanoscale HE‐MOFs at room temperature. However, the disadvantages of this method include the time‐consuming process required for growing HE‐MOFs crystals, and the need for precise control of reaction time to regulate crystal structure and morphology. Ma et al. first reported the fabrication of high‐entropy Prussian blue analogs (HE‐PBA) by integrating Mn, Co, Ni, Fe, and Cu ions into the framework structure via the co‐precipitation method (**Figure** **4**a).^[^ ^69^ ^]^ Although the classification of PBA as MOF is a topic of debate, we include it in this review, since the functionality and morphology of PBA compounds resemble classical MOFs. Although not purely organic, C≡N acts as a linker between metal nodes, forming an ordered and porous 3D network structure, which is the hallmark of MOF structures. The typical chemical formula of PBAs can be described as A_x_M[Fe(CN)_6_]_y_□_m_·nH_2_O, where A, M, and □ represent alkaline metal ions, transition metal ions, and vacancies, respectively.^[^ ^82^ ^]^ The 3D framework structure of PBAs is constructed by alternating coordination between M and Fe metal sites with the cyanide group, where high‐entropy M sites coordinate with nitrogen, and Fe sites coordinate with carbon.^[^ ^83^ ^]^ In this work, the HE‐PBA showed a single‐phase cubic structure without any impurity (Figure 4b). Based on the results of inductively coupled plasma‐optical emission spectroscopy (ICP‐OES) and thermogravimetric analysis (TGA), the chemical formula of HE‐PBA was determined as Na_1.19_(Fe_0.2_Mn_0.2_Ni_0.2_Cu_0.2_Co_0.2_)[Fe(CN)_6_]_0.79_□_0.21_·1.16H_2_O, which illustrates its equimolar proportion of metal species, in accordance with the characteristic of HEMs. Additionally, the X‐ray photoelectron spectroscopy (XPS) analysis not only demonstrated the co‐existence of multiple elements in HE‐PBA but also provided the chemical state of each metal species (Figure 4c). Recently, applying the co‐precipitation method, a series of Prussian white analogs (PW) with up to six metal cations were fabricated by He et al.^[^ ^68^ ^]^ As shown in Figure 4d, it is evident that six metal ions (Mn, Fe, Ni, Cu, Co, and Cd) collectively share the high‐entropy metal sites, while Fe_1_ ions occupy the Fe sites, which constructs the framework structure of high‐entropy PW (HE‐PW) through coordinating with the cyanide group. The XRD pattern and Rietveld refinement result revealed that the obtained HE‐PW exhibits a monoclinic structure with the space group of P2_1_/n (Figure 4e). Moreover, the scanning electron microscope (SEM) images illustrated that HE‐PW is composed of cubic nanoparticles, and EDS maps revealed a relatively uniform distribution of multiple cations at the high‐entropy metal sites (Figure 4f,g).

### 3.3 Mechanochemical Synthesis

**Figure 5:**
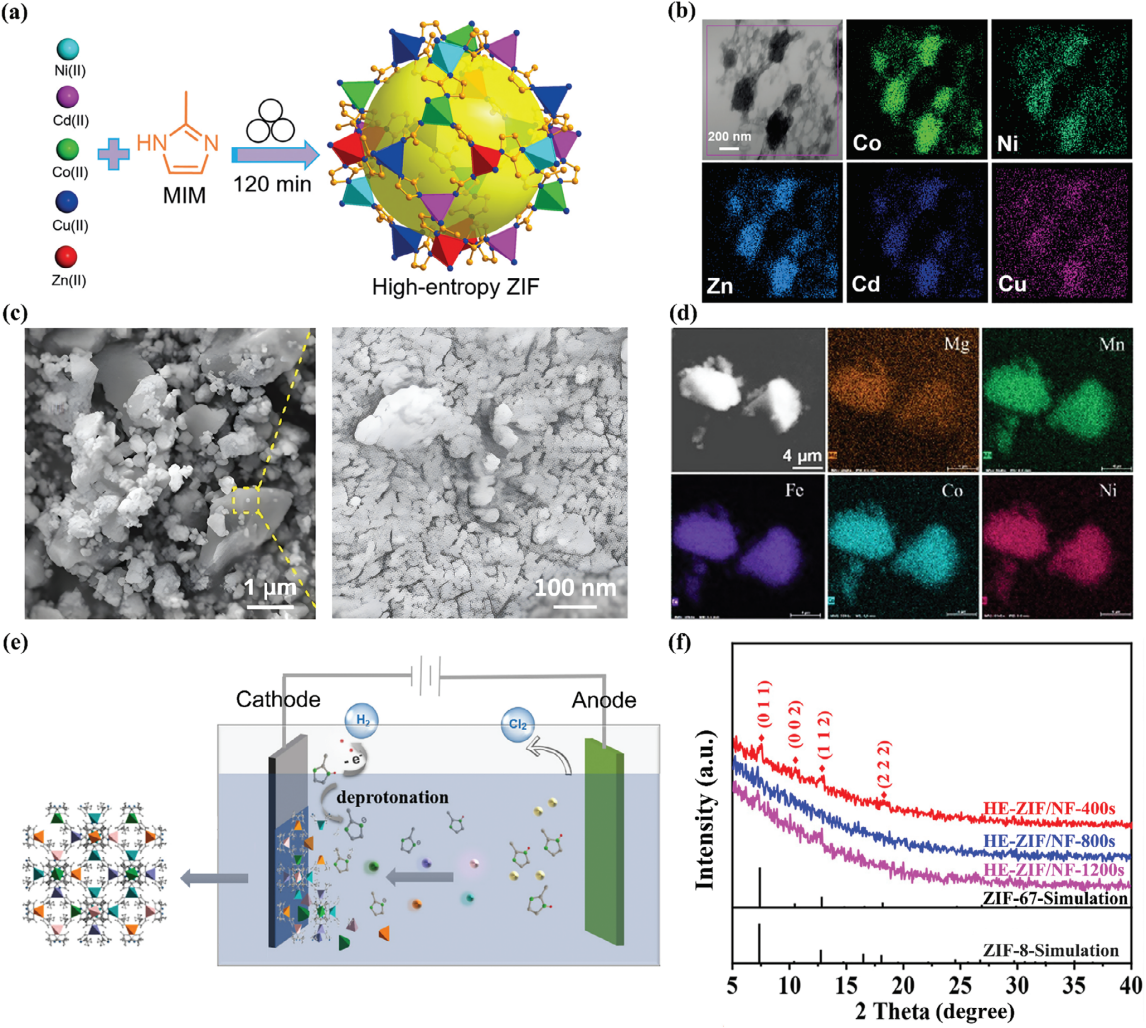
a) Schematic illustration of the synthesis and b) EDS maps of HE‐ZIF. Reproduced with permission.^[^ ^80^ ^]^ Copyright 2019, Wiley‐VCH. c) SEM images and d) EDS maps of MgMnFeCoNi‐HEPBA. Reproduced with permission.^[^ ^85^ ^]^ Copyright 2021, Elsevier. e) Schematic illustration of the electrodeposition process of HE‐ZIF. f) XRD patterns of HE‐ZIF, ZIF‐67‐simulation, and ZIF‐8‐simulation. Reproduced with permission.^[^ ^75^ ^]^ Copyright 2023, Elsevier.

A further method for fabricating HE‐MOFs is through a combined mechanochemistry and wet chemistry synthetic route. A notable advantage of this method is the substantial reduction in reaction time. During the ball milling process, the generated mechanical and frictional energy can provide local short‐range heating to the reactants, promoting fast diffusion of metal ions and leading to the formation of HE‐MOFs. However, such intense friction is also a drawback of this approach, as it creates numerous defects in HE‐MOFs and results in lower crystallinity. Xu et al. prepared a high‐entropy zeolitic imidazolate framework (HE‐ZIF) through ball milling CdO, CuO, ZnO, Ni(OAc)_2_, Co(OAc)_2_, and excess 2‐methylimidazole at room temperature for 120 min (**Figure** **5**a).^[^ ^80^ ^]^ According to the results of EDS mapping (Figure 5b), it is clear that Cd^2+^, Cu^2+^, Zn^2+^, Ni^2+^, and Co^2+^ are homogeneously distributed in HE‐ZIF. In addition, compared to conventional Cd‐ZIF, the diffraction peaks of HE‐ZIF illustrated a higher angle shift, which provides evidence for the successful incorporation of metal ions with smaller ionic radii, such as Zn^2+^ (0.60 Å), Co^2+^ (0.58 Å), into the HE‐ZIF structure. Similarly, Wang et al. reported the synthesis of HE‐MOF by ball milling procedure.^[^ ^84^ ^]^ A mixture of (CH_3_COO)_2_Co⋅4H_2_O, (CH_3_COO)_2_Ni⋅4H_2_O, WCl_6_, RuCl_3_, H_2_MoO_4_, and 1,4‐benzene dicarboxylic acid was completely converted into HE‐MOF after 180 min ball milling. The resulting HE‐MOF demonstrated a smooth nanosheet structure with uniform elemental dispersion, as proved by SEM images and EDS maps. Jiang et al. also reported the successful synthesis of MgMnFeCoNi‐HEPBA via a two‐step method (ball milling five transition metal salts and potassium ferricyanide, followed by water rinsing).^[^ ^85^ ^]^ The XRD pattern of MgMnFeCoNi‐HEPBA revealed its single‐phase structure with the space group of Fm‐3 m, which is well‐indexed to the cubic phase of PBA. However, according to the SEM images and EDS maps (Figure 5c,d), it is clear that while MgMnFeCoNi‐HEPBA illustrates a uniform elemental distribution, its morphology appears as an amorphous bulk structure with numerous surface cracks. This result suggests that ball milling promotes rapid interaction between organic ligands and metal ions, but also leads to increased defects in MgMnFeCoNi‐HEPBA.

### 3.4 Electrodeposition Synthesis

In addition, the electrodeposition process has also been investigated for synthesizing HE‐MOFs. Specifically, when high overpotentials are applied, the electrodeposition reaction is controlled by the mass transfer of metal ions, which eliminates the differences in coordination abilities of various metal ions with organic ligands. As illustrated in Figure 5e, Dong et al. reported the preparation of Ni foam‐supported HE‐ZIF by electrodeposition method and achieved the regulation of morphology and catalytic properties of the deposits through rational design of electrodeposition parameters.^[^ ^75^ ^]^ Based on transmission electron microscopy (TEM) and EDS images, the morphology of the optimized HE‐ZIF can be described as amorphous nanoparticles with a homogeneous distribution of Zn^2+^, Co^2+^, Ni^2+^, Mn^2+^, and Fe^3+^. Additionally, four diffraction peaks were detected in the XRD pattern of HE‐ZIF, which are consistent with the standard patterns of ZIF‐67‐simulation and ZIF‐8‐simulation, illustrating the formation of single‐phase structure (Figure 5f).

## 4 HE‐MOFs for Catalysis Applications

### 4.1 Water Splitting

The electrocatalytic water splitting for hydrogen production is considered as a promising strategy in the energy sector, due to its inherent advantages of simplicity, carbon‐free emissions, and high efficiency.^[^ ^86^, ^87^ ^]^ Generally, hydrogen and oxygen are generated by two half‐reactions: the hydrogen evolution reaction (HER) occurring at the cathode and the oxygen evolution reaction (OER) occurring at the anode. However, in practical applications, the sluggish kinetics caused by the multiple‐electron transfer in HER and OER severely limits the overall efficiency of water splitting. To address this challenge, Pt and RuO_2_/IrO_2_ have been employed as efficient catalysts for HER and OER, respectively. Nevertheless, the high cost and low reserves of noble metal‐based catalysts hinder their large‐scale industrial application. Therefore, developing economical and highly efficient catalysts is essential. Preferably, these catalysts should possess exceptional catalytic activity and maintain stability under acidic or alkaline conditions during operation. Furthermore, high conductivity is also desired to facilitate rapid electron transfer. Recently, HE‐MOFs and their derivatives, composed of abundant and cost‐effective transition metals, have attracted significant attention in the field of water splitting (**Table** **1**). Specifically, integrating various elements into high‐entropy lattices endows HE‐MOFs with a unique and diverse surface structure, with each metal ion serving as an individual catalytic site, collectively contributing to the overall catalytic activity. Additionally, the lattice distortion and redistribution of electronic structure originating from the high‐entropy strategy also provide HE‐MOFs with some distinctive features, such as lower electron transfer resistance, making them a promising alternative to noble metal catalysts.

**Table 1:** List of all HE‐MOFs and their derivatives for water splitting.

Catalyst	Precursor	Application	Substrate	Overpotential	Tafel slope	References
NiCoFeZnMo‐MOF	–	OER	Ni foam	254 mV at 50 mA cm^−2^ in 1.0 m KOH	61 mV dec^−1^	[81]
Fe_0.2_Co_0.2_Ni_0.2_Mn_0.2_Cu_0.2_‐MOF	–	OER	Glassy carbon	315 mV at 10 mA cm^−2^ in 1.0 m KOH	49 mV dec^−1^	[72]
MnFeCoNiCu‐MOF	–	OER	Glassy carbon	245 mV at 10 mA cm^−2^ in 1.0 m KOH	54 mV dec^−1^	[61]
MnFeCoNiZn‐ZIF	–	OER	Ni foam	259 mV at 10 mA cm^−2^ in 1.0 m KOH	37 mV dec^−1^	[75]
CoNiCuMnAl/C	CoNiCuMnAl‐MOF	OER	Carbon paper	290 mV at 10 mA cm^−2^ in 1.0 m KOH	68.8 mV dec^−1^	[88]
ZnFeNiCuCoRu‐O	ZnCoFeNiCu‐MOF	OER	Glassy carbon	170 mV at 10 mA cm^−2^ in 1.0 m KOH	56 mV dec^−1^	[89]
(MnFeCoNiCu)S_2_	MnFeCoNiCu‐MOF	OER	Ni foam	221 mV at 10 mA cm^−2^ in 1.0 m KOH	54.4 mV dec^−1^	[90]
(W_28_Ni_24_Co_24_Mo_17_Ru_7_)PO_x_/C	CoNiWRuMo‐MOF	HER	Glassy carbon	40 mV at 10 mA cm^−2^ in 0.5 m H_2_SO_4_	36 mV dec^−1^	[84]

#### 4.1.1 Oxygen Evolution Reaction

**Figure 6:**
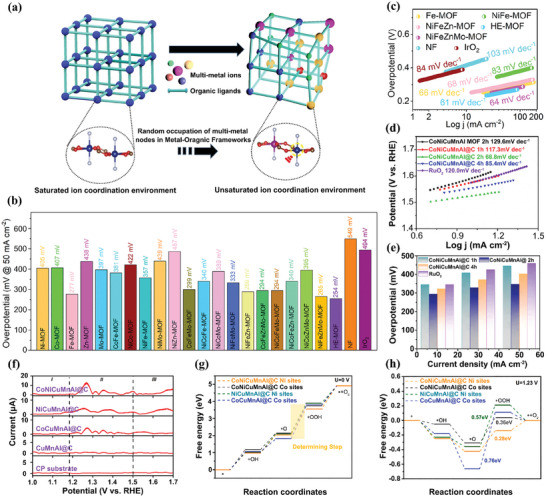
a) Schematic illustration of modulating the metal sites coordination environment in HE‐MOF by the introduction of multi‐metal ions with various radii. Reproduced with permission.^[^ ^72^ ^]^ Copyright 2022, Wiley‐VCH. b) The overpotential testing at a current density of 50 mA cm^−2^ in 1.0 m KOH electrolyte and c) Tafel plots of unary, binary, ternary, quaternary, quinary MOFs, IrO_2_, and pure Ni foam. Reproduced with permission.^[^ ^81^ ^]^ Copyright 2022, American Chemical Society. d) Tafel plots and e) comparison of overpotential at 10, 30, and 50 mA cm^−2^ of different HEAs/C and RuO_2_ catalysts in 1.0 m KOH electrolyte. f) The 6th harmonic FTACV curves of CoNiCuMnAl@C, NiCuMnAl@C, CoCuMnAl@C, CuMnAl@C, and carbon paper at 10 Hz and 100 mV. Standard free energy diagram of OER process at g) 0 V and h) 1.23 V of CoNiCuMnAl@C Ni sites, CoNiCuMnAl@C Co sites, NiCuMnAl@C Ni sites, and CoCuMnAl@C Co sites. Reproduced with permission.^[^ ^88^ ^]^ Copyright 2022, Elsevier.

Yao et al. synthesized HE‐MOF as a working electrode for OER through the coordination of Mn, Fe, Ni, Co, and Cu metal ions with organic ligands of 2,5‐thiophenedicarboxylic acid (TDC) and 4,4‐bipyridine (BIPY).^[^ ^72^ ^]^ Benefiting from the integration of multiple cations, lattice distortion was induced in HE‐MOF, which modifies the coordination environments at catalytic metal sites and generates oxygen vacancies (**Figure** **6**a), thus leading to enhanced binding energy between the catalytic surface and intermediates, as well as improved electrocatalytic activity compared to conventional Co‐TDC‐BIPY MOF. As a result, the HE‐MOF required a lower overpotential of 315 mV to achieve the current density of 10 mA cm^−2^ and delivered a smaller Tafel plot of 49 mV dec^−1^. Furthermore, they also explored the individual impact of each metal in HE‐MOF for OER. The results demonstrated that Co atoms exhibit higher catalytic activity compared to other transition metals in HE‐MOF, which is consistent with the previous report.^[^ ^91^ ^]^ This can be ascribed to the increase in high‐valence Co originating from multiple elements integration, thereby affecting its adsorption‐desorption strength toward oxygen intermediates and ultimately achieving outstanding OER performance.

Similarly, using the solvothermal method, Xu et al. fabricated a series of MOFs with diverse metal compositions using 2,6‐naphthalenedicarboxylate tetrahydrate ligand.^[^ ^81^ ^]^ The catalytic performance for OER of these MOFs were thoroughly investigated in 1 m KOH electrolyte. In this study, utilizing Ni foam as the substrate, the HE‐MOF displayed a distinct 2D nanosheet array structure with tens of nanometer‐sized pores between adjacent sheets. This unique microstructure is conducive to the formation of highly exposed active metal sites and accelerates mass transfer during OER. Moreover, the high‐entropy approach induces additional structural vacancies, causing distortion in atomic arrangements and redistribution of charge density, which reduces electron transfer resistance and enhances electrical conductivity. Additionally, incorporating multi‐metal into HE‐MOF significantly increases the number of active centers and enhances intermediates adsorption, effectively decreasing the reaction overpotential and achieving remarkable electrocatalytic performance. Furthermore, the associated effect among the various metal elements can greatly improve the stability and reusability. Consequently, compared with other MOFs and the commercial catalyst IrO_2_, the HE‐MOF delivered the smallest overpotential of 254 mV at 50 mA cm^−2^ (Figure 6b), the lowest Tafel slope of 61 mV dec^−1^ (Figure 6c), and good electrochemical stability with negligible drop after 100 h of testing, which strongly implies improved electrochemical performance via the high‐entropy strategy.

In addition to direct use as electrocatalysts for OER, HE‐MOFs have been employed as precursors to fabricate diverse HEMs, such as HEAs, HEOs, HESs, and HEPs, which also exhibit great potential in catalyzing water splitting.^[^ ^84^, ^88^, ^89^, ^90^ ^]^ For example, Wang et al. devised a MOF‐templated strategy to prepare a series of nanosized CoNiCuMnAl/C (HEAs/C) by adjusting the stirring time of the precursor solution.^[^ ^88^ ^]^ The OER activity of as‐obtained HEAs/C was examined in an O_2_‐saturated 1 m KOH electrolyte and compared to the commercial RuO_2_ catalyst. As presented in Figure 6d, an ultra‐low Tafel slope value of 68.8 mV dec^−1^ was achieved by CoNiCuMnAl@C 2 h, superior to those of CoNiCuMnAl@C 1 h, CoNiCuMnAl@C 4 h, and RuO_2_. Moreover, the CoNiCuMnAl@C 2 h displayed the smallest overpotential at various current densities among all the prepared samples, indicating its outstanding electrocatalytic activity (Figure 6e). According to the analysis of Fourier‐transformed alternating current voltammetry (FTACV) and density functional theory (DFT) calculations (Figure 6f–h), the author concluded that the exceptional catalytic performance of CoNiCuMnAl@C 2 h can be explained by the synergistic effect of multi‐metallic sites. In fact, Ni and Co metal sites are catalytically active centers, significantly reducing the energy barrier of the rate‐determining step in the OER process, while Cu, Mn, and Al metal sites act to accelerate electron transfer. Additionally, the outer thin carbon shell generated by the MOF‐templated method can protect the catalyst in alkaline solutions, allowing CoNiCuMnAl@C 2 h to maintain long‐term stability even under 200 mA cm^−2^.

**Figure 7:**
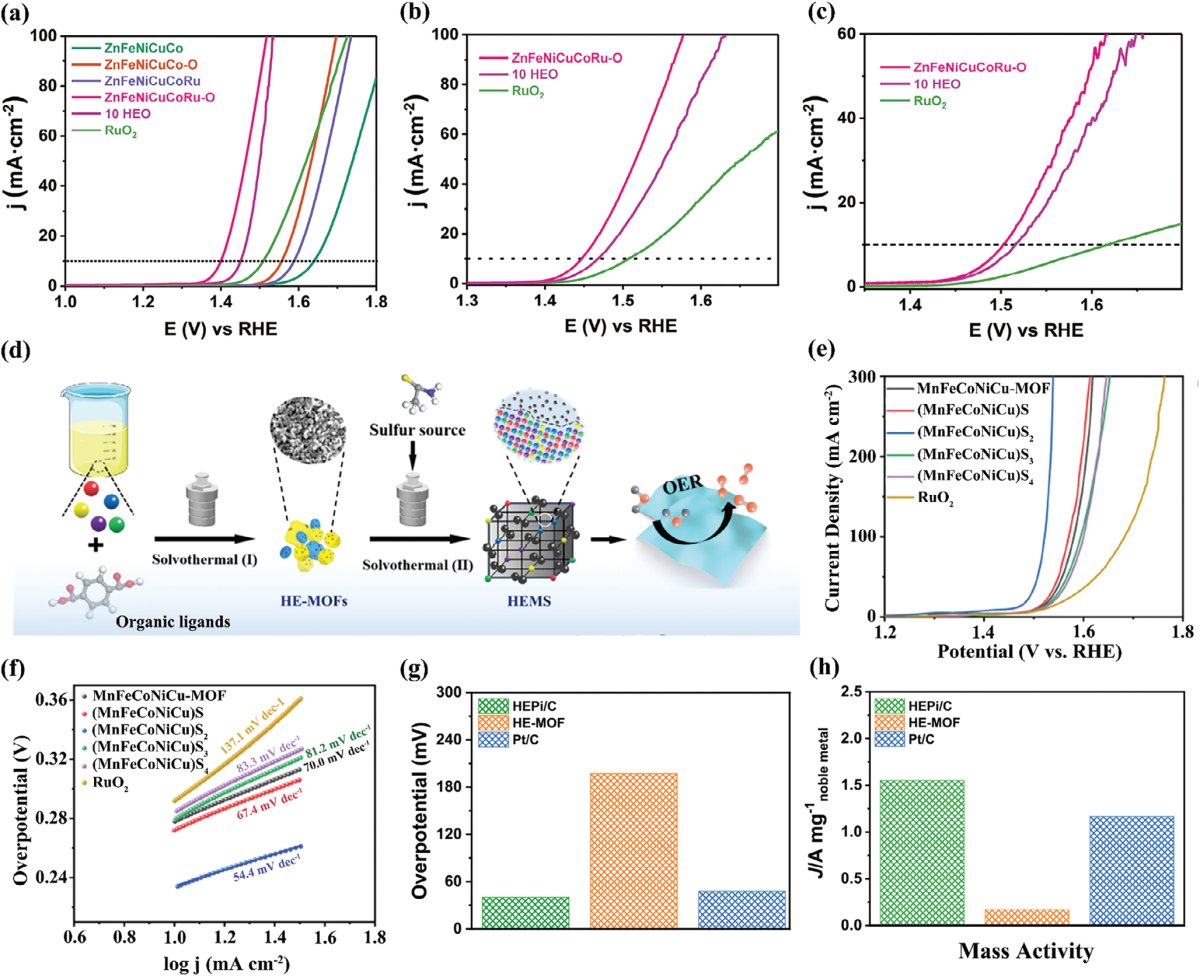
Linear sweep voltammetry (LSV) polarization curves of ZnFeNiCuCoRu‐O, quinary HEO, denary HEO, and RuO_2_ at a current density of 10 mA cm^−2^ in a) 1.0 m KOH, b) 0.5 m H_2_SO_4_, and c) 1.0 m PBS electrolyte, respectively. Reproduced with permission.^[^ ^89^ ^]^ Copyright 2023, Wiley‐VCH. d) Schematic illustration of the synthesis of HEMS nanoparticles via a two‐step solvothermal method. e) LSV curves and f) the corresponding Tafel curves of MnFeCoNiCu‐MOF, (MnFeCoNiCu)S_x_, and RuO_2_ in N_2_‐saturated 1.0 m KOH. Reproduced with permission.^[^ ^90^ ^]^ Copyright 2022, American Chemical Society. g) Overpotential of HEPi/C, HE‐MOF, and Pt/C at 10 mA cm^−2^ in 0.5 m H_2_SO_4_ electrolyte. h) Mass activity of HEPi/C, HE‐MOF, and Pt/C at an overpotential of 50 mV. Reproduced with permission.^[^ ^84^ ^]^ Copyright 2022, Elsevier.

Miao et al. reported the synthesis of hollow‐structured and polyhedron‐shaped ZnFeNiCuCoRu‐O using MOF as a sacrificial template via the ion exchange method.^[^ ^89^ ^]^ When applied as catalyst for OER, the ZnFeNiCuCoRu‐O demonstrated impressive catalytic activity in full pH range, with an overpotential of 170, 215, and 270 mV at a current density of 10 mA cm^−2^ in 1.0 m KOH (pH ≈ 14), 0.5 m H_2_SO_4_ (pH ≈ 0), and 1 m PBS (pH ≈ 7) aqueous solutions, respectively, surpassing its quinary and denary counterparts as well as the commercial RuO_2_ (**Figure** **7**a–c). Additionally, owing to its unique hollow structure, ZnFeNiCuCoRu‐O illustrated both a large electrochemically active surface area (87.88 cm^2^) and exceptional chemical stability. Consequently, an extremely low Tafel slope value of 56 mV dec^−1^ was detected in ZnFeNiCuCoRu‐O, lower than those of ZnFeNiCuCo‐O (65 mV dec^−1^), ZnCoFeNiCuVCrMnMoRu‐O (58 mV dec^−1^), and commercial RuO_2_ (87 mV dec^−1^), which indicates its fast OER kinetics. Furthermore, in stark contrast to the rapid decay of RuO_2_, ZnFeNiCuCoRu‐O demonstrated superior long‐term stability in an extreme reaction environment with only a slight decay of 7% observed during 30 h testing. This was evidenced by its well‐preserved polyhedral shape and randomly dispersed elements.

Utilizing HE‐MOF as the precursor, high‐entropy (MnFeCoNiCu)S_2_ (HEMS) was synthesized via a hydrothermal method (Figure 7d).^[^ ^90^ ^]^ The HEMS was evaluated as an electrocatalyst for OER in N_2_‐saturated 1.0 m KOH solution. Electrochemical analysis revealed that the HEMS displayed the highest electrocatalytic activity, with an overpotential of 221 mV at 10 mA cm^−2^, surpassing commercial RuO_2_ (295 mV) and other quinary metal sulfides (Figure 7e). As shown in Figure 7f, it also demonstrated the smallest Tafel slope of 54.4 mV dec^−1^, indicating enhanced reaction kinetics. Additionally, it was found that the HEMS exhibited excellent stability and durability with a near‐zero current decay (≈2.4%) after 12 h of continues electrolysis. The outstanding electrocatalytic performance of HEMS can be explained by three key factors. First, the application of the high‐entropy strategy not only generates abundant active sites but also stabilizes the electronic structure of the obtained materials. Second, the nanoscale HEMS exhibits a large specific surface area, which enhances the interaction between the catalyst and electrolyte, thereby facilitating accelerated mass transport during the OER. Finally, the cocktail effect generated by multi‐element integration can reduce the free energy of the system, thus leading to a more stable framework structure, while the presence of Fe and Ni can rapidly generate a passivation layer to prevent further oxidation.

#### 4.1.2 Hydrogen Evolution Reaction

Similar to the aforementioned studies on utilizing HE‐MOFs and their derivatives as catalysts for OER, researchers also investigated their catalytic performance for HER in acidic conditions. For instance, Ru, Ni, Co, Mo, W‐based high‐entropy phosphate/carbon (HEPi/C) composite derived from HE‐MOF was fabricated through a mechanochemical synthesis method followed by phosphorylation treatment.^[^ ^84^ ^]^ The HEPi/C was tested as an electrocatalyst for HER in 0.5 m H_2_SO_4_ electrolyte, and compared to HE‐MOF precursor and commercial Pt/C. As shown in Figure 7g,h, the HEPi/C composite demonstrated a lower HER overpotential of 40 mV at the current density of 10 mA cm^−2^ and a higher mass activity of 1.6 A mg_noble metal_^−1^ at the overpotential of 50 mV compared to the commercial Pt/C catalyst (48 mV and 1.2 A mg_noble metal_^−1^). The remarkable electrocatalytic performance of HEPi/C can be explained by the synergistic effect of Ru, Ni, Co, Mo, and W. Moreover, the carbon‐coated nanosheet structure, combined with the high surface area of HEPi/C, significantly enhances conductivity and generates a higher number of active sites for HER.

A noble‐metal‐free MnNiCoZn‐based HE‐MOF was investigated by Qi et al.,^[^ ^92^ ^]^ and reported for the first time as a photocatalyst for hydrogen evolution. Strictly speaking, the HE‐MOF described in this study does not fulfill the quantitative definition for a HE‐MOF and is more accurately classified as a multi‐metal MOF or ME‐MOF due to its limited Δ*S* (<1.5 R). However, compared to single‐metal MOFs, this multi‐metal MOF exhibited superior photocatalytic HER activity. The rationale behind such exceptional performance could be attributed to the incorporation of multiple metal elements, which enables a more favorable band alignment between the organic linkers and multi‐metal nodes, facilitating fast electron transfer. This work highlights the significant research potential of multi‐metal MOFs in HER and also provides strong evidence supporting the superiority of the entropic effect.

### 4.2 Nitrogen Fixation

Electrochemical fixation of nitrogen (N_2_) to ammonia (NH_3_) is widely acknowledged as a highly favorable approach for NH_3_ synthesis.^[^ ^93^, ^94^ ^]^ This method utilizes earth‐abundant water and nitrogen as raw materials, with renewable electricity serving as the driving force, which possesses several advantages over the conventional Haber‐Bosch process, including low cost, environmental friendliness, and high efficiency.^[^ ^95^ ^]^ Nonetheless, like water splitting, highly active and ultra‐stable catalysts are also required to overcome the large energy barriers of multiple‐electron transfer processes, such as the six‐electron transfer for nitrogen reduction reaction (NRR) and four‐electron transfer for OER, in electrochemical fixation of N_2_ before they can reach the market.

**Figure 8:**
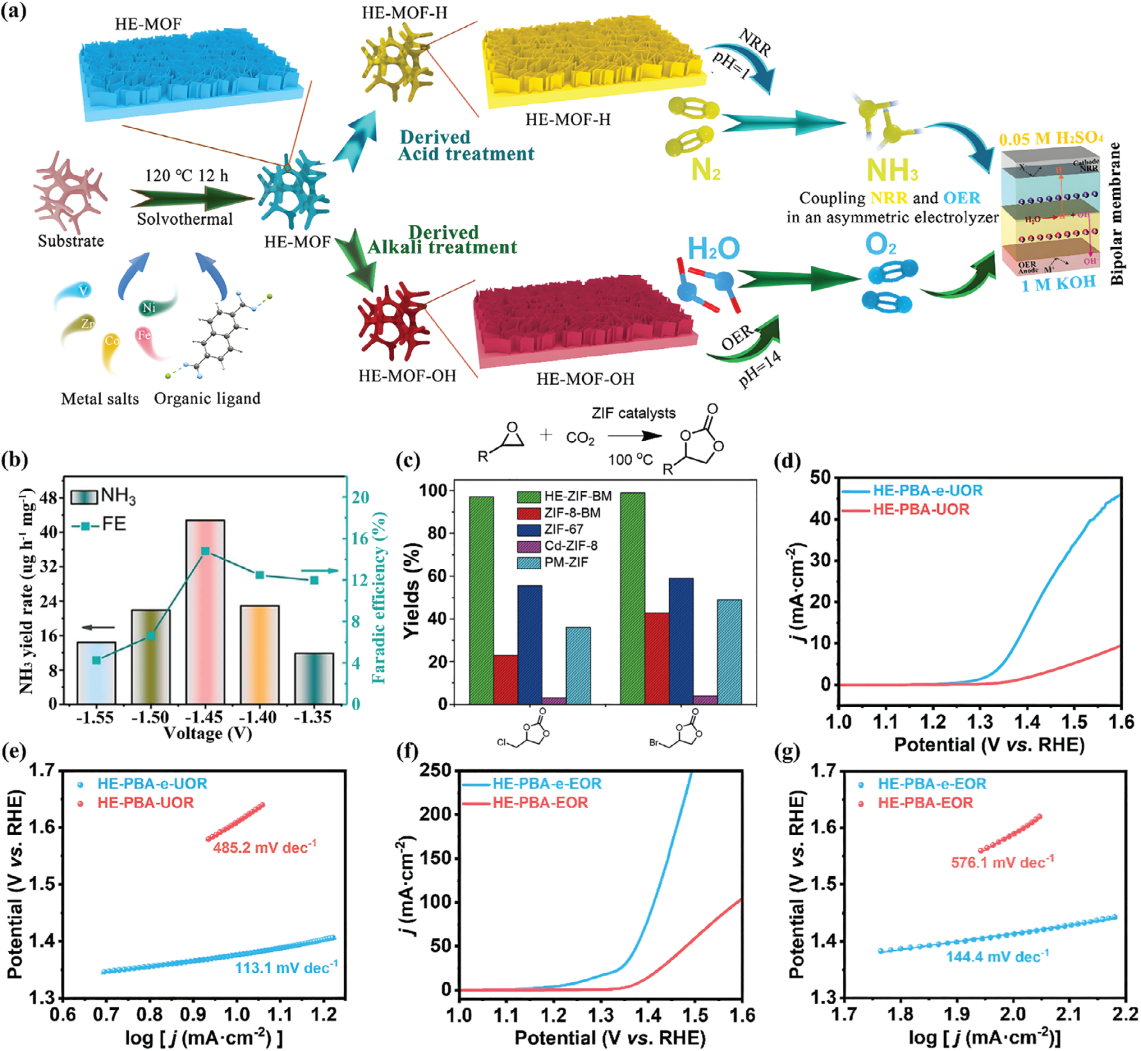
a) Schematic illustration of asymmetric acidic/alkaline N_2_ electrochemical fixation accelerated by HE‐MOF derivatives. b) NH_3_ yield rates and Faradaic efficiencies of HE‐MOF and its derivatives at different applied potentials. Reproduced with permission.^[^ ^74^ ^]^ Copyright 2023, Wiley‐VCH. c) Yields of two cyclic carbonates obtained from the cycloaddition of CO_2_ with corresponding epoxides catalyzed by HE‐ZIF and its counterparts. Reproduced with permission.^[^ ^80^ ^]^ Copyright 2019, Wiley‐VCH. Electrocatalytic performance of HE‐PBA and HE‐PBA‐e toward UOR. d) LSV polarization curves, e) Tafel plots. Electrocatalytic performance of HE‐PBA and HE‐PBA‐e toward EOR. f) LSV polarization curves, g) Tafel plots. Reproduced with permission.^[^ ^64^ ^]^ Copyright 2023, American Chemical Society.

Recently, Sun et al. designed NiCoFeZnV‐based HE‐MOF using a chemical deposition method and prepared its derivatives (HE‐MOF‐H and HE‐MOF‐OH) through a facile acid/alkaline treatment.^[^ ^74^ ^]^ Subsequently, employing the generated HE‐MOF‐H and HE‐MOF‐OH as catalysts for NRR and OER in different pH electrolytes, they successfully constructed an asymmetric N_2_ electrochemical fixation device using the bipolar membranes in a flow‐type cell (**Figure** **8**a). As presented in Figure 8b, the obtained device displayed impressive electrocatalytic activity, with a high NH_3_ yield rate of 42.76 µg h^−1^ mg^−1^ and Faraday efficiencies of 14.75% at −1.45 V. Additionally, extremely high energetic efficiency of 2.59% was achieved by this asymmetric configuration, surpassing the value of its symmetric counterparts (0.18% for acidic electrolyte and 0.59% for alkaline electrolyte). The author proposed that the exceptional performance can be ascribed to the optimized electronic structure, hierarchical porous nanosheet morphology, and synergistic effect of multi‐metallic sites of HE‐MOF. Furthermore, the usage of flow‐type cells promoted the mass transfer at the gas‐liquid‐solid interface, facilitating the fast reaction kinetics.

### 4.3 Carbon Dioxide Conversion

Catalytic cycloaddition reaction of carbon dioxide (CO_2_) with epoxides is a highly promising route for effectively converting CO_2_ into high‐value cyclic carbonates while alleviating the greenhouse effect.^[^ ^96^, ^97^ ^]^ Nevertheless, as a typical nonpolar molecule, the high energy barrier associated with the C═O bond in CO_2_ hinders its efficient conversion.^[^ ^98^ ^]^ Consequently, developing highly active and robust catalysts is urgently needed.

Xu et al. synthesized HE‐ZIF with a uniform distribution of cations through ball grinding at ambient temperature.^[^ ^80^ ^]^ The catalytic activity of HE‐ZIF, single‐metal ZIFs (including Zn‐ZIF (ZIF‐8), Co‐ZIF (ZIF‐67), and Cd‐ZIF (Cd‐ZIF‐8)), and a physical mixture of these three single‐metal ZIFs (PM‐ZIF) was evaluated under 10 bar CO_2_ pressure at 100 °C for 8 h. As shown in Figure 8c, HE‐ZIF revealed excellent catalytic performance in the CO_2_ cycloaddition reaction, achieving 99.0% yield for 2‐(bromomethyl)oxirane and 97.2% yield for 2‐(chloromethyl)oxirane, respectively, surpassing the value catalyzed by both single‐metal ZIFs and PM‐ZIF. The improved catalytic performance of HE‐ZIF is a benefit of the rich Lewis acid sites generated by the integration of multiple cations, highlighting the intrinsic advantages of the high‐entropy strategy.

### 4.4 Electrooxidation of Urea and Ethanol

Direct urea fuel cells (DUFCs) and direct ethanol fuel cells (DEFCs) have stimulated intense research interest due to their theoretically high energy density, high efficiency, and low pollution.^[^ ^99^, ^100^ ^]^ However, developing novel electrocatalysts to facilitate the sluggish anodic multi‐electron transfer reactions, specifically the urea oxidation reaction (UOR) and ethanol oxidation reaction (EOR), is crucial for achieving effective energy conversion devices. While Pt is recognized as a promising electrocatalyst due to its exceptional catalytic activity toward both the UOR and EOR, its scarcity and high cost severely hinder large‐scale industrial applications. Therefore, exploring noble‐metal‐free catalytic systems with both high catalytic activity and cost‐effectiveness is receiving significant attention.

Xu et al. developed NH_3_⋅H_2_O‐etched HE‐PBA (HE‐PBA‐e) through a simple co‐precipitation method followed by an etching treatment.^[^ ^64^ ^]^ Compared to pristine HE‐PBA, HE‐PBA‐e surprisingly demonstrated a modified electronic structure with a higher proportion of Co^3+^ and Ni^3+^ species, which are known to be effective catalytical centers. Moreover, a much‐enhanced electrochemical active surface area was detected in HE‐PBA‐e, which significantly promotes the exposure of active metal sites as well as their contact with the electrolyte. Consequently, when tested as a catalyst for UOR and EOR, the HE‐PBA‐e illustrated superior catalytic activity (Figure 8d,f) and better reaction kinetics (Figure 8e,g). Additionally, the electrochemical durability of HE‐PBA‐e was also evaluated for UOR and EOR, showing no obvious decline after operating at a current density of 10 mA cm^−2^ for 80 and 75 h, respectively, indicating its long‐term stability as a multifunctional catalyst.

### 4.5 Aerobic Oxidation of Benzyl Alcohol

Selective aerobic oxidation of benzyl alcohol (BAL) is an important reaction for the production of valuable oxygenates.^[^ ^101^, ^102^ ^]^ However, the existing catalytic systems often suffer from insufficient active sites and low catalytic efficiency, which hinder their practical applications. Therefore, it is highly paramount to develop a suitable catalyst with the features of high activity, long‐term stability, high efficiency, and low cost.

**Figure 9:**
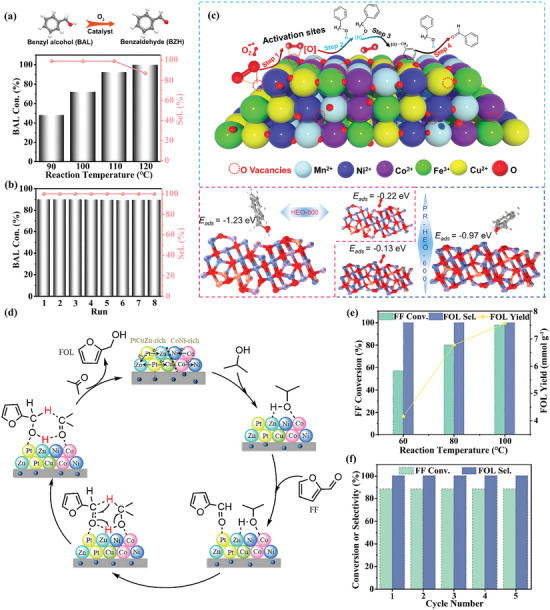
a) The catalytic performance of HEO‐600 for BAL selective aerobic oxidation at different temperatures and b) reusability during the catalytic procedure. c) Plausible mechanism for the selective aerobic oxidation of BAL to BZH for HEO‐600 and models of BAL and O_2_ adsorbed on HEO‐600 and its counterpart. Reproduced with permission.^[^ ^67^ ^]^ Copyright 2023, American Chemical Society. d) The reaction pathways for CTH of FF using PtCoNiCuZn@NC‐600. e) The catalytic performance of PtCoNiCuZn@NC‐600 at different temperatures and f) recycling experiments of PtCoNiCuZn@NC‐600 for the CTH of FF to FOL. Reproduced with permission.^[^ ^105^ ^]^ Copyright 2023, Elsevier.

Zhang et al. reported a MOF‐templated synthesis of flower‐like HEO (HEO‐600) with nanosheet structure for BAL oxidation via a two‐step method.^[^ ^67^ ^]^ In this work, benefiting from rational compositional and structural design, HEO‐600 exhibited abundant oxygen vacancies and exposed sufficient multiple metal sites, which modulate its electronic structure as well as the microchemical environment, thus facilitating high activity, fast kinetics, and robust cycling stability in BAL oxidation. As a result, the HEO‐600 demonstrated outstanding catalytic performance, achieving a high selectivity of 99.9% and a yield of 92.3% toward benzaldehyde (BZH) at the temperature of 110 °C (**Figure** **9**a), along with good sustainability and economic viability, showing no obvious decline in activity even after 8 cycles (Figure 9b). Additionally, as shown in Figure 9c, theoretical simulations further confirmed that oxygen vacancies play a crucial role in activating reactants, thereby significantly promoting the formation of reactive oxygen species and facilitating subsequent oxidation processes.

### 4.6 Catalytic Transfer Hydrogenation of Furfural

Catalytic transfer hydrogenation (CTH) is a highly effective and eco‐friendly method for converting renewable biomass resources into high‐value products, including high‐energy density fuels and functional chemicals.^[^ ^103^, ^104^ ^]^ In practical applications, organic molecules like alcohols, ammonia borane, and formic acid commonly serve as hydrogen donors to reduce the chemical bonds with the assistance of catalysts. Therefore, the activity and selectivity of catalysts are critical factors that determine the efficient conversion of biomass resources in hydrogenation reactions.

Liu et al. prepared a nitrogen‐doped carbon‐supported HEA (PtCoNiCuZn@NC‐600) by utilizing HE‐MOF as a sacrificial template via pyrolysis.^[^ ^105^ ^]^ The as‐obtained PtCoNiCuZn@NC‐600 was used, for the first time, as the catalyst in the CTH of furfural (FF) to furfuryl alcohol (FOL) and delivered a near‐perfect catalytic performance (Figure 9d). Notably, as presented in Figure 9e, the PtCoNiCuZn@NC‐600 achieved 100% conversion of FF and 100% selectivity toward FOL under room pressure at the temperature of 100 °C. Additionally, the PtCoNiCuZn@NC‐600 illustrated excellent cycling stability, as evidenced by the consistent FF conversion and FOL selectivity over five consecutive cycles (Figure 9f). Such impressive catalytic performance of PtCoNiCuZn@NC‐600 can be ascribed to the cocktail effect generated by multi‐metal doping, which reduces the amount of precious metal while still maintaining good catalytic activity. Furthermore, the author proposed that the uniform elemental distribution of PtCoNiCuZn@NC‐600 nanoparticles and the regulation of strong acid sites on the surface also play important roles in enhancing the catalytic behavior.

### 4.7 Summary of HE‐MOFs for Catalysis Applications

The above reports suggest that HE‐MOFs and their derivatives show promise as catalysts for various applications. Compared to traditional MOFs, the lattice distortion caused by integrating multiple elements can modulate the electronic structure of high‐entropy metal sites and create abundant oxygen vacancies. This enhances the interactions between metal sites and intermediates as well as facilitates fast kinetics. Furthermore, the cocktail effect of multi‐metallic sites can significantly improve both the catalytic activity and stability of HE‐MOFs. Consequently, benefiting from these advantages, HE‐MOFs demonstrate exceptional catalytic performance, even surpassing noble‐metal‐based catalysts in oxygen evolution and hydrogen evolution reactions. However, current reports on the application of HE‐MOFs in catalysis are quite limited and mainly focus on emphasizing the improvement of catalytic performance, severely lacking in‐depth exploration of the mechanism of how the high‐entropy strategy enhances catalytic activity, such as its effects on reaction pathways and energy barriers. Additionally, more systematic studies combining experimental and theoretical calculations are needed to identify the role of each individual element in HE‐MOFs during the catalytic process, facilitating rational composition design to achieve the desired performance.

## 5 HE‐MOFs for Energy Storage Applications

### 5.1 Rechargeable Batteries

Rechargeable batteries, as the representative of electrochemical energy storage technology, have been widely utilized due to their high efficiency, environmental friendliness, and low cost.^[^ ^106^, ^107^, ^108^ ^]^ As essential components of the battery system, the storage capacity and structural stability of electrode materials significantly impact the performance of rechargeable batteries. Therefore, designing advanced electrode materials is of immense importance for achieving desirable electrochemical performance. HE‐MOFs, possessing the merits of abundant active energy storage centers and stable framework structures, have shown great potential in various battery systems for electrochemical energy storage (**Table** **2**).

**Table 2:** List of all HE‐MOFs for rechargeable batteries.

HE‐MOFs	Space group	Application	Performance	Reference
Na_1.19_(Fe_0.2_Mn_0.2_Ni_0.2_Cu_0.2_Co_0.2_)[Fe(CN)_6_]_0.79_	Fm‐3m	SIBs/Cathode	94% capacity retention after 150 cycles at 100 mA g^−1^	[69]
Na_1.26_(Mn_0.4_Fe_0.15_Ni_0.15_Cu_0.15_Co_0.15_)[Fe(CN)_6_]_0.81_	Fm‐3m	SIBs/Cathode	90% capacity retention after 200 cycles at 100 mA g^−1^	[27]
Na_1.41_(Mn_0.32_Fe_0.11_ Co_0.28_Ni_0.32_Cu_0.32_)[Fe(CN)_6_]	Fm‐3m	SIBs/Cathode	95% capacity retention after 10 000 cycles at 1500 mA g^−1^	[77]
Na_1.65_(Mn_0.4_Fe_0.12_Ni_0.12_Cu_0.12_Co_0.12_Cd_0.12_)[Fe(CN)_6_]_0.92_	P2_1_/n	SIBs/Cathode	75% capacity retention after 100 cycles at 30 mA g^−1^	[68]
K_1.29_(Mn_0.2_Fe_0.2_Co_0.21_Ni_0.19_Cu_0.21_)[Fe(CN)_6_]_0.85_	P2_1_/n	SIBs/Cathode	82.8% capacity retention after 300 cycles at 100 mA g^−1^	[62]
Na_1.70_(Fe_0.2_Mn_0.2_Co_0.2_Ni_0.2_Cu_0.2_)[Fe(CN)_6_]_0.98_	P2_1_/n	SIBs/Cathode	79.6% capacity retention after 1000 cycles at 100 mA g^−1^	[63]
K_1.59_(Mn_0.30_Co_0.19_Ni_0.07_Fe_0.22_Cu_0.22_)[Fe(CN)_6_]_0.62_	P2_1_/n	ZIBs/Cathode	82% capacity retention after 100 cycles at 100 mA g^−1^	[109]
Na_1.19_(Zn_0.23_Mn_0.19_Ni_0.19_Cu_0.21_Co_0.18_)[Fe(CN)_6_]_0.81_	Fm‐3m	ASIBs/Cathode	87% capacity retention after 1000 cycles at 1 C (full cell with NaTi_2_(PO_4_)_3_@C anode)	[70]
Co_0.2_Ni_0.2_Cu_0.2_Mn_0.2_Zn_0.2_‐PBA	Fm‐3m	Li–S/Cathode	570.9 mAh g^−1^ after 200 cycles at 0.1 C	[110]

#### 5.1.1 Sodium‐Ion Batteries

**Figure 10:**
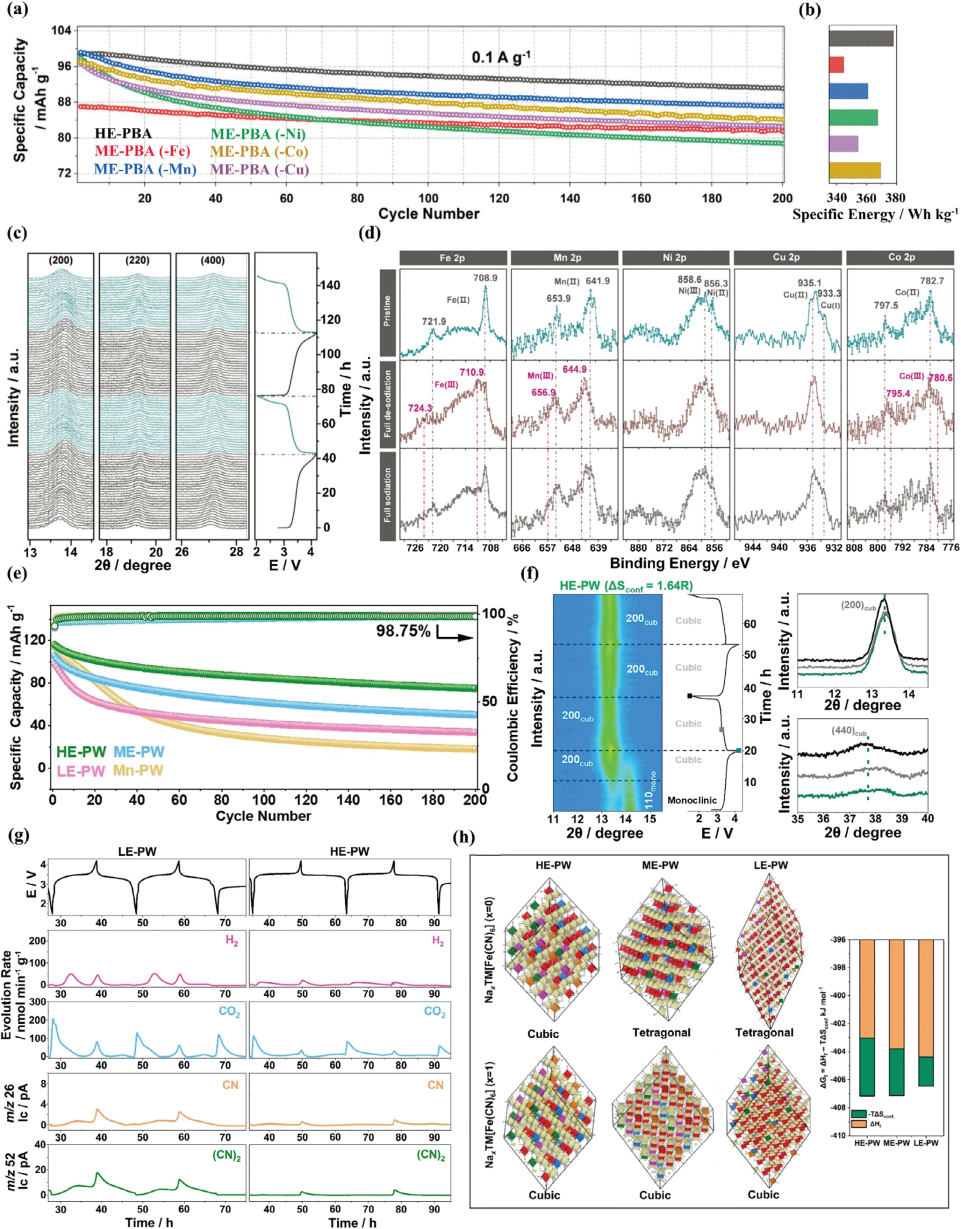
a) Galvanostatic cycling performance at 0.1 A g^−1^ and b) comparison of specific energies for HE‐PBA and different medium‐entropy PBAs. c) Operando XRD characterization of the electrochemical extraction/insertion of Na^+^ from/into HE‐PBA. d) Ex situ XPS analysis of HE‐PBA‐based electrodes before/after cycling. Reproduced with permission.^[^ ^69^ ^]^ Copyright 2021, Wiley‐VCH. e) Specific discharge capacity versus cycle number at 30 mA g^−1^ of HE‐PW, ME‐PW, LE‐PW, and Mn‐PW cathodes. f) Operando XRD characterization of HE‐PW cathode over the initial two cycles. g) In situ gas evolution profiles for HE‐PW and LE‐PW cathodes as obtained via DEMS. h) The calculated crystal structure of HE‐PW, ME‐PW, and LE‐PW in a fully charged state (top) and partially discharged state (down), as well as their Gibbs free energy of formation in a partially charged state at 298K. Reproduced with permission.^[^ ^68^ ^]^ Copyright 2023, Wiley‐VCH.

Ma et al. developed Na_1.19_(Fe_0.2_Mn_0.2_Ni_0.2_Cu_0.2_Co_0.2_)[Fe(CN)_6_]_0.79_□_0.21_ (HE‐PBA) through a facile co‐precipitation method and used it as cathode material in sodium‐ion batteries (SIBs) for the first time.^[^ ^69^ ^]^ As illustrated in **Figure** **10**a, compared with the medium‐entropy PBAs counterparts, the HE‐PBA displayed an improved Na^+^ storage capacity (an initial capacity of 100 mAh g^−1^ at a current density of 0.1 A g^−1^) and superior cycling stability (capacity retention of 94% after 150th cycles). Moreover, benefiting from the increased mean discharge voltage, the HE‐PBA achieved the highest gravimetric energy density (378 Wh kg^−1^) among all prepared samples (Figure 10b). According to the results of operando XRD test and ex situ XPS analysis (Figure 10c,d), the author concluded that such remarkable electrochemical performance of HE‐PBA can be attributed to its robust structure and synergistic effect. Specifically, unlike conventional PBAs, which undergo complex phase transitions during the sodiation/de‐sodiation processes, the HE‐PBA consistently maintained a cubic structure with almost zero strain throughout the operation. In addition, the ex situ XPS results revealed that Mn^2+/3+^, Fe^2+/3+^, Co^2+/3+^, and Cu^1+/2+^ serve as the primary redox centers in HE‐PBA, while the presence of redox inactive Ni^2+^ helps stabilize the framework structure. The cocktail effect arising from the interaction between these cations plays a crucial role in achieving both high specific capacity and long‐term stability.

Very recently, He et al. synthesized a series of PWs via a co‐precipitation approach at room temperature and explored their electrochemical performance as cathodes in SIBs.^[^ ^68^ ^]^ The as‐obtained Na_1.65_(Mn_0.4_Fe_0.12_Ni_0.12_Cu_0.12_Co_0.12_Cd_0.12_)[Fe(CN)_6_]_0.92_□_0.08_ (HE‐PW) cathode exhibited superior electrochemical performance compared to its medium‐entropy (ME‐), low‐entropy (LE‐), and conventional manganese (Mn‐) PW counterparts (Figure 10e). For instance, after 100 cycles at the current density of 30 mA g^−1^, the capacity retention for HE‐PW, ME‐PW, LE‐PW, and Mn‐PW were 75%, 60%, 42.5%, and 25%, respectively. Based on operando XRD tests (Figure 10f), the author revealed that the exceptional electrochemical performance of HE‐PW is a benefit of the increased configurational entropy of the system, which effectively suppresses the phase transition to monoclinic or tetragonal phases and allows for the maintenance of a symmetrical cubic phase during the reversible cycles. In addition, the gas evolution of HE‐PW and LE‐PW was quantitatively compared by the in situ gas analysis via differential electrochemical mass spectrometry (DEMS). The results (Figure 10g) indicated that the disordered composition of HE‐PW is beneficial in mitigating gas evolution caused by the side reactions during cycles, leading to lower structural degradation. Furthermore, DFT calculations were conducted to determine the Gibbs free energies of formation for each considered phase of HE‐PW, ME‐PW, and LE‐PW at different charge‐discharge states, aiming to theoretically investigate the influence of configurational entropy on their stability (Figure 10h). Unlike ME‐PW and LE‐PW, the lowest energy state of HE‐PW consistently corresponds to the cubic phase with high symmetry and stability, regardless of whether it is in the fully de‐sodiated state or sodiated state. These computational results are in mutual agreement with the experimental data and demonstrate, at the level of atomic lattice, that the high‐entropy approach contributes to the formation of a robust lattice structure.

#### 5.1.2 Aqueous Zinc/Sodium‐Ion Batteries

**Figure 11:**
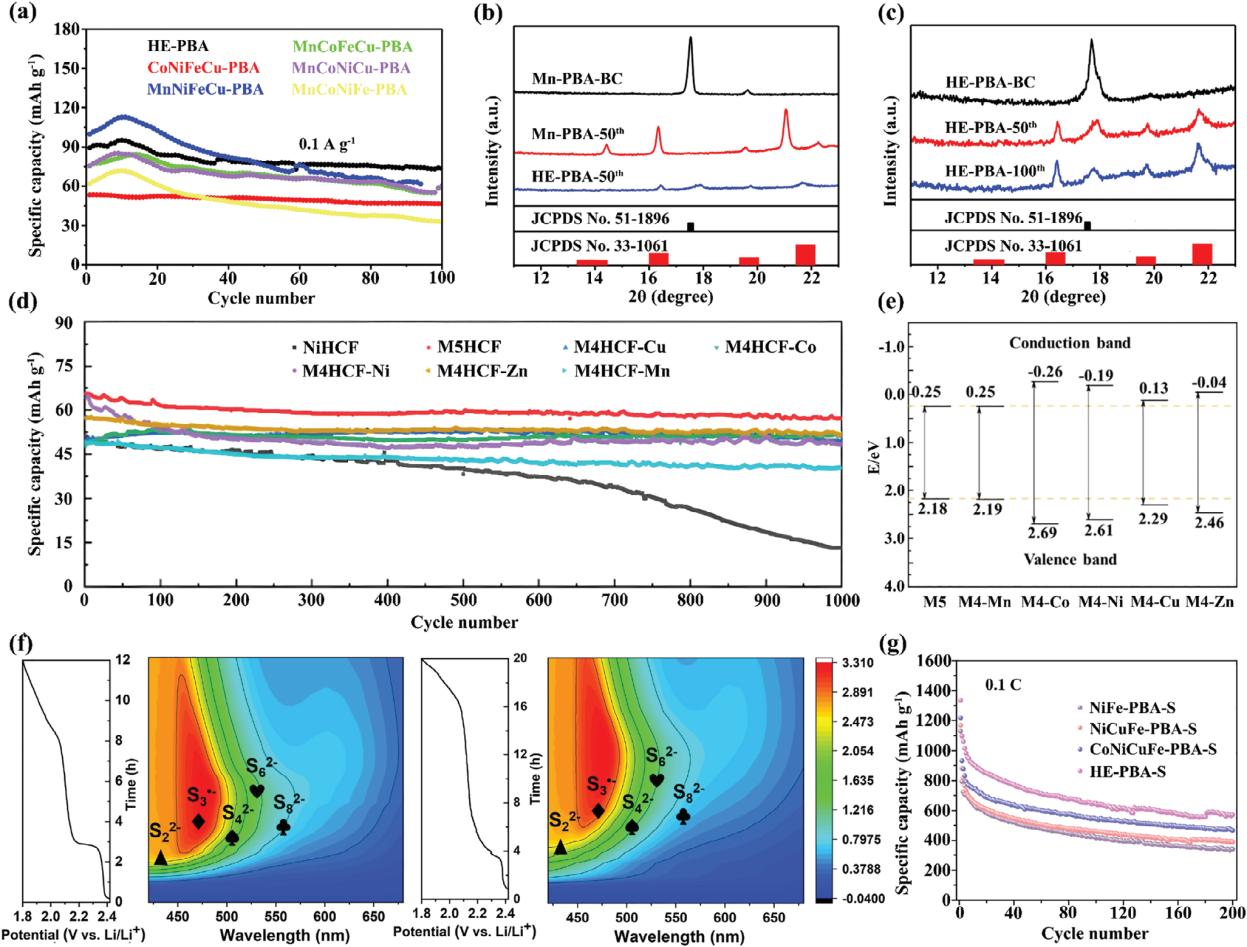
a) Cycling performance of HE‐PBA and different quaternary PBAs at 0.1 A g^−1^. b) XRD patterns of HE‐PBA and Mn‐PBA electrodes after 50th cycles. c) XRD patterns of HE‐PBA electrode after different cycles. Reproduced with permission.^[^ ^109^ ^]^ Copyright 2022, Springer Nature. d) Cycling performance of M5HCF and different M4HCFs cathodes in full cells at 1 C. e) Schematic diagram of the band edges for M5HCF and different M4HCFs. Reproduced with permission.^[^ ^70^ ^]^ Copyright 2023, Royal Society of Chemistry. f) Contour maps of in situ UV–Vis spectra and the corresponding discharge profiles of NiFe‐PBA‐S (left) and HE‐PBA‐S (right). g) Cycling performance of NiFe‐PBA‐S, NiCuFe‐PBA‐S, CoNiCuFe‐PBA‐S, and HE‐PBA‐S cathode at 0.1 C over 200 cycles. Reproduced with permission.^[^ ^110^ ^]^ Copyright 2022, Wiley‐VCH.

HE‐MOFs were also investigated as cathode materials for zinc‐ion batteries (ZIBs). Through integrating Mn, Fe, Ni, Co, and Cu metal ions into the same N‐coordinated sites, Xing et al. fabricated K_1.59_(Mn_0.30_Co_0.19_Ni_0.07_Fe_0.22_Cu_0.22_)[Fe(CN)_6_]_0.62_ (HE‐PBA).^[^ ^109^ ^]^ As shown in **Figure** **11**a, in stark contrast to the rapid capacity degradation in different quaternary PBAs, the HE‐PBA delivered a high specific capacity of ≈90 mAh g^−1^ with a superior capacity retention of 82% at a current density of 0.1 A g^−1^ after 100 cycles. The excellent electrochemical performance of HE‐PBA can be explained by the cocktail effect of multi‐element doping, which mitigates the uncontrollable phase transition caused by the dissolution of Mn^2+^ and the Jahn‐Teller effect during the cycles (Figure 11b,c). Additionally, based on the ex situ XPS results, it is explained that some of the cations are involved in the uptake and removal of Zn^2+^, while the non‐redox‐active cations are responsible for stabilizing the lattice structure and enhancing the cycling life.

Similarly, the Mn, Co, Ni, Cu, Zn‐based Na_1.19_(Zn_0.23_Mn_0.19_Ni_0.19_Cu_0.21_Co_0.18_)[Fe(CN)_6_]_0.81_□_0.19_ (M5HCF) was explored as cathode material and assembled with NaTi_2_(PO_4_)_3_@C to form aqueous sodium‐ion full batteries (ASIBs).^[^ ^70^ ^]^ The obtained full cell displayed a superior rate performance with an average capacity of 75 mAh g^−1^ at 0.5 C and an excellent cycling performance with a capacity retention of 87% after 1000 cycles at 1 C (Figure 11d). The DFT calculations reveal that the application of the high‐entropy strategy enables M5HCF to possess a smaller bandgap than its different quaternary counterparts (M4HCF), which reduces the difficulty associated with the electron excitation from the valence band to the conduction band and leads to faster diffusion kinetics (Figure 11e). Moreover, by analyzing the XPS spectra of M5HCF before and after cycling, the author observed a decrease in the proportion of high‐valence transition metal ions with strong oxidation abilities in cycled M5HCF electrodes. Such a decrease consequently reduces the number of sodium insertion sites, which maybe one of the reasons for the capacity degradation of M5HCF electrode during long‐term cycling.

#### 5.1.3 Lithium–Sulfur Batteries

In addition to the aforementioned reports, HE‐MOFs have been investigated as catalyst materials to improve the cycling stability of lithium–sulfur (Li–S) batteries. For example, Du et al. successfully prepared a series of PBAs, ranging from binary to quinary compositions, to serve as sulfur host materials for Li–S batteries via a two‐step method (i.e., co‐precipitation to synthesis PBAs followed by melt‐diffusion to loading S).^[^ ^110^ ^]^ According to in situ UV–Vis analysis and visual lithium polysulfides (LiPS) adsorption tests, the author concluded that benefiting from the homogeneously distributed cations, when implemented as cathode, HE‐PBA not only acts as a catalyst to facilitate the fast conversion between S_8_ and Li_2_S_2_/Li_2_S, but also exhibits a strong affinity for soluble LiPS, thereby alleviating the notorious shuttle effect (Figure 11f). As a result, the HE‐PBA‐S showed remarkable electrochemical performance with a specific capacity of 570.9 mAh g^−1^ after 200 cycles at 0.1 C (Figure 11g), which encompasses the value of its counterparts (467, 389.8, and 339.5 mAh g^−1^ for CoNiCuFe‐PBA‐S, NiCuFe‐PBA‐S, and NiFe‐PBA‐S, respectively).

**Figure 12:**
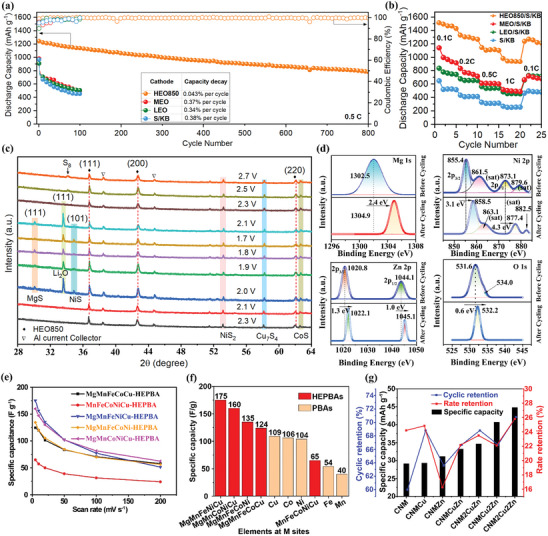
Comparison of a) long‐term cycling stability at 0.5 C and b) rate performance at various C rates of HEO850/S/KB, MEO/S/KB, LEO/S/KB, and S/KB cells. c) Ex situ XRD measurement on the HEO850/S/KB cathode at various discharging and charging conditions. d) High‐resolution XPS spectra of Mg 1s, Ni 2p, Zn 2p, and O 1s before and after 100 cycles at 0.5 C. Reproduced with permission.^[^ ^111^ ^]^ Copyright 2023, Wiley‐VCH. e) Specific capacitance of HEPBA series derived from CVs at different scan rates. f) Summary of specific capacitances for HEPBAs and single component PBAs with a scan rate of 5 mV s^−1^. Reproduced with permission.^[^ ^85^ ^]^ Copyright 2021, Elsevier. g) Specific capacity, rate retention, and cyclic retention of different ternary, quaternary, and quinary PBAs. Reproduced with permission.^[^ ^65^ ^]^ Copyright 2023, Royal Society of Chemistry.

Recently, utilizing multi‐element MOF as the precursor, Raza et al. developed a novel low‐temperature approach to synthesize (Ni_0.2_Co_0.2_Cu_0.2_Mg_0.2_Zn_0.2_)O (HEO850) as a host material for Li–S batteries.^[^ ^111^ ^]^ As shown in **Figure** **12**a,b, the obtained cathode (HEO850/S/KB) delivered an ultra‐stable cycling performance (with a negligible capacity decay of 0.043% per cycle at 0.5 C after 800 cycles) and a superior rate capability (with the discharge capacity reaching up to 1513, 1330, 1147, and 985 mAh g^−1^ at 0.1, 0.2, 0.5, and 1 C, respectively), which are greater than the corresponding values of its medium‐entropy and low‐entropy counterparts (MEO/S/KB and LEO/S/KB). Based on ex situ XPS and ex situ XRD analysis (Figure 12c,d), it is concluded that the active sites (such as Mg, Ni, Zn, and O) in HEO850/S/KB are essential for chemically anchoring polysulfides, which effectively suppresses soluble polysulfides shuttle and results in exceptional electrochemical performance.

### 5.2 Supercapacitors

Supercapacitors (SCs) with the merits of ultrafast charge–discharge rate, wide operating temperature range (−10 to 80 °C), and long cycle life (>10^5^ cycles), are regarded as one of the most promising and efficient energy storage technologies, which have attracted numerous attentions in high‐power‐demanding devices.^[^ ^112^, ^113^ ^]^ The main limitation of the practical applications of SCs is related to their inherently low energy density.^[^ ^114^ ^]^ Consequently, the crucial factor in unleashing their complete potential lies in the design and development of high‐performance novel electrode materials. Recently, as a new class of HEMs, HE‐MOFs have been reported for SCs applications.

Jiang et al. reported a series of HEPBAs with five highly dispersed metal ions synthesized at room temperature by combining mechanochemistry with wet chemistry.^[^ ^85^ ^]^ Among as‐fabricated HEPBAs, the MgMnFeNiCu‐HEPBA delivered impressive supercapacitor performance with a specific capacitance of 175 F g^−1^ at 5 mV s^−1^, outperforming other HEPBAs with different compositions and all conventional PBAs (Figure 12e,f). Such exceptional performance can be ascribed to the uniform distribution of diverse metal ions at the atomic level, as well as the cocktail effect achieved through the optimized composition. Moreover, the defects in MgMnFeNiCu‐HEPBA improve capacitance performance through the creation of micro‐porous structures, while the presence of abundant adsorbed and interstitial water promotes Grotthuss proton conduction by forming a hydrogen‐bonding network. In another study, Nguyen et al. produced a series of ternary, quaternary, and quinary PBAs for supercapacitor application.^[^ ^65^ ^]^ Benefiting from Cu/Zn co‐doping, the HEPBA exhibited smaller particle sizes, increased vacancies, and larger surface area. As a result, the HEPBA showed a specific capacitance of 336 F g^−1^ at a current density of 1 A g^−1^, along with excellent long‐term stability (Figure 12g).

### 5.3 Summary of HE‐MOFs for Energy Storage Applications

The aforementioned studies confirm the tremendous research potential of HE‐MOFs and their derivatives in electrochemical energy storage systems. Applying the high‐entropy strategy to HE‐MOFs introduces multiple metal ions into their framework structure, creating abundant redox‐active sites and thereby enhancing their electrochemical performance. Moreover, the presence of non‐redox active cations in HE‐MOFs plays a significant role in stabilizing the crystal structure during reversible cycles, resulting in excellent long‐term stability. Despite some progress in the use of HE‐MOFs as cathode materials for rechargeable batteries, their capacity has not yet reached a satisfactory level. Meanwhile, research on the development of HE‐MOFs as anode materials is completely absent, indicating that there is still a long way to go before constructing a full HE‐MOFs battery. In addition, although the high‐entropy strategy can mitigate the Jahn‐Teller effect in HE‐PBAs to some extent, the inevitable degradation of electrode materials over long cycles will continue to affect battery lifespan. Hence, research on high‐entropy MOFs containing six or more metal components is necessary to explore the influence of increased configurational entropy on alleviating the Jahn‐Teller effect. Finally, in future research, nano‐morphology design should be applied to HE‐MOFs to regulate their contact area with the electrolyte and thereby enhance their electronic conductivity. This advancement will enable them to achieve high‐energy density and high‐power density at the same time, thus meeting the requirements of high‐power demanding devices.

## 6 Theoretical and Computational Techniques for HE‐MOFs

**Figure 13:**
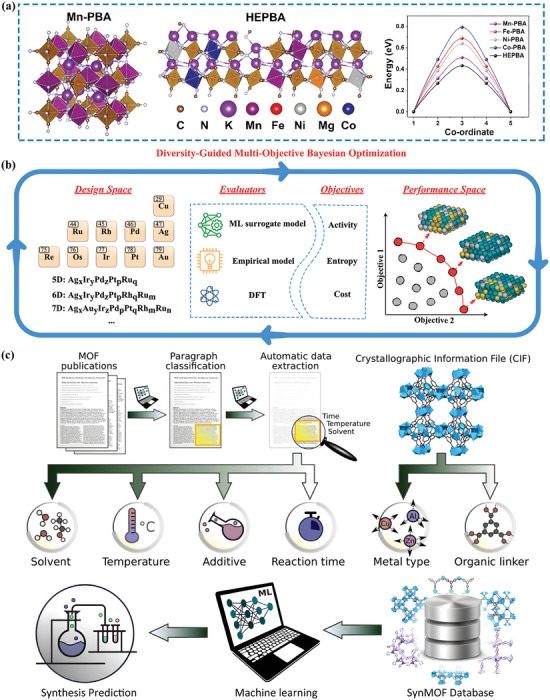
a) The optimized structures of Mn‐PBA and HE‐PBA, and their corresponding formation energy. Reproduced with permission.^[^ ^115^ ^]^ Copyright 2023, Wiley‐VCH. b) Schematic illustration of the framework for discovering HEA electrocatalysts for oxygen reduction reactions. Reproduced with permission.^[^ ^120^ ^]^ Copyright 2024, American Chemical Society. c) Schematic illustration of the ML model for accelerating the discovery of new MOFs and predicting the corresponding synthesis conditions. Reproduced with permission.^[^ ^121^ ^]^ Copyright 2022, Wiley‐VCH.

Despite the rapid advancements in experimental research on HE‐MOFs, most studies have limited the optimization to a space of five to six elements, with selection often based on existing simple single‐metal MOFs or chemical intuition. To achieve efficient exploration of the vast design space of HE‐MOFs, theoretical and computational approaches are essential. As shown in **Figure** **13**a, several studies have utilized DFT calculations to reveal the underlying mechanisms connecting the high‐entropy strategy to the outstanding catalytic or energy storage performance.^[^ ^48^, ^115^, ^116^ ^]^ However, due to the compositional complexity and highly disordered lattice structures of high‐entropy systems, as well as the time‐consuming calculation process, DFT is generally applicable to property calculations of HE‐MOFs with specific compositions but provides only limited guidance for experimental screening across larger compositional landscapes. Recent advances in machine learning (ML), however, offer a promising pathway to accelerate the discovery of novel multi‐component high‐entropy systems.^[^ ^117^, ^118^, ^119^ ^]^ Xu et al. proposed a data‐efficient multi‐objective Bayesian optimization framework driven by the ML surrogate model for discovering novel catalysts composed of higher‐dimensional constituent elements (Figure 13b).^[^ ^120^ ^]^ The designed method can simultaneously optimize catalytic activity, mixing entropy, and manufacturability within a vast design space, ultimately presenting a series of promising candidates. Although HEAs were chosen as a showcase in this work, it still demonstrate great potential for application in the rational design of HE‐MOFs. Similarly, Luo et al. presented a MOF discovery strategy using ML models to directly predict the synthesis conditions based on MOF crystal structures (Figure 13c), which provides a highly valuable tool for the advancement of the MOF community.^[^ ^121^ ^]^ Therefore, although there are currently no reports on the direct use of ML models for HE‐MOFs, it is quite certain that ML can also be applied to design multi‐metal HE‐MOFs within a higher‐dimensional composition space and optimize their physicochemical properties for catalysis and energy applications.

## 7 Summary and Outlook

HE‐MOFs, as an emerging member of HEMs, have illustrated remarkable performance in the research areas of catalysis as well as energy storage and conversion due to their inherent merits derived from high‐entropy strategy, including abundant active sites, exceptional porosity, robust framework structure, and tunable chemical properties. However, the development of HE‐MOFs is in its nascent stages, there is still much research work that needs to be performed to achieve further progress in this field.

Despite we have briefly introduced the design principles for the synthesis strategies of HE‐MOFs, and summarized the commonly used methods based on the existing reports, developing a novel large‐scale synthesis approach remains a major limitation in realizing the practical applications of HE‐MOFs. In our opinion, to efficiently exploit the cocktail effect, lattice distortion, and other features of HE‐MOFs, the synthetic method requires a bottom‐up construction of HE‐MOFs to achieve maximum homogeneous mixing of multiple elements at the atomic level. On the other hand, the newly designed route should possess the characteristics of low cost, environmental friendliness, simple equipment, and easy procedures.

Beyond synthesis, we notice that there seems to be a relatively poor understanding of the completely disordered arrangement of various elements within the HE‐MOFs lattices and the relationship between the role of individual components with excellent performance. In most of the reported literature on HE‐MOFs, the uniform dispersion of multiple elements is typically demonstrated through EDS characterization and ICP‐OES analysis. However, it should be noted that these techniques can only exhibit the elemental distribution at the microscale and reveal the relative content of different metal species, respectively, without providing definitive evidence of atomic‐scale homogeneous mixing. Additionally, the commonly used characterization techniques like in situ XRD and in situ XPS are insufficient for revealing the fundamental relationship between rational compositional design and the excellent performance of HE‐MOFs. To overcome these limitations, higher precision characterization techniques such as atom probe tomography and synchrotron radiation X‐ray technology should be employed to accurately assess the elemental distributions and identify the conformational relationship between high‐entropy structure and performance in future studies.

It is also challenging that there are virtually no reports focusing on purely theoretical computations and computational investigations of HE‐MOFs, despite the rapid development of experimental studies on them. Hence, it is highly paramount to employ theoretical calculation methods, such as DFT calculations and ab initio molecular dynamics, and establish suitable models of HE‐MOFs to deepen our understanding of the intrinsic mechanisms behind high‐entropy strategies. Furthermore, advanced computational simulation approaches, including artificial intelligence and ML, are also needed to explore the relationship between high‐entropy components and the corresponding properties of HE‐MOFs by inputting and analyzing large amounts of data. These results can offer innovative guidance for optimizing the composition of HE‐MOFs in a more cost‐effective and time‐saving approach to achieve the desired properties.

## Conflict of Interest

The authors declare no conflict of interest.
